# Leveraging process mining for modeling progression trajectories in amyotrophic lateral sclerosis

**DOI:** 10.1186/s12911-023-02113-7

**Published:** 2023-02-02

**Authors:** Erica Tavazzi, Roberto Gatta, Mauro Vallati, Stefano Cotti Piccinelli, Massimiliano Filosto, Alessandro Padovani, Maurizio Castellano, Barbara Di Camillo

**Affiliations:** 1 Department of Information Engineering, University of Padova, Via Gradenigo 6/b, 35131 Padua, Italy; 2 Department of Clinical and Experimental Sciences, University of Brescia, Viale Europa 11, 25121 Brescia, Italy; 3 School of Computing and Engineering, University of Huddersfield, Huddersfield, HD1 3DH UK; 4 NeMO-Brescia Clinical Center for Neuromuscular Diseases, Via Paolo Richiedei 16, 25064 Gussago, Italy; 5 Unit of Neurology, ASST Spedali Civili, Piazzale Spedali Civili 1, 25123 Brescia, Italy; 6 Department of Comparative Biomedicine and Food Science, University of Padova, Agripolis, Viale dell’Università, 16, 35020 Legnaro, Italy

**Keywords:** Amyotrophic lateral sclerosis, Progression trajectories, Process mining, Process discovery, Patient stratification, Prognosis

## Abstract

**Background:**

Amyotrophic Lateral Sclerosis (ALS) is a neurodegenerative disease whose spreading and progression mechanisms are still unclear. The ability to predict ALS prognosis would improve the patients’ quality of life and support clinicians in planning treatments. In this paper, we investigate ALS evolution trajectories using Process Mining (PM) techniques enriched to both easily mine processes and automatically reveal how the pathways differentiate according to patients’ characteristics.

**Methods:**

We consider data collected in two distinct data sources, namely the Pooled Resource Open-Access ALS Clinical Trials (PRO-ACT) dataset and a real-world clinical register (ALS–BS) including data of patients followed up in two tertiary clinical centers of Brescia (Italy). With a focus on the functional abilities progressively impaired as the disease progresses, we use two Process Discovery methods, namely the Directly-Follows Graph and the CareFlow Miner, to mine the population disease trajectories on the PRO-ACT dataset. We characterize the impairment trajectories in terms of patterns, timing, and probabilities, and investigate the effect of some patients’ characteristics at onset on the followed paths. Finally, we perform a comparative study of the impairment trajectories mined in PRO-ACT versus ALS–BS.

**Results:**

We delineate the progression pathways on PRO-ACT, identifying the predominant disabilities at different stages of the disease: for instance, 85% of patients enter the trials without disabilities, and 48% of them experience the impairment of Walking/Self-care abilities first. We then test how a spinal onset increases the risk of experiencing the loss of Walking/Self-care ability as first impairment (52% vs. 27% of patients develop it as the first impairment in the spinal vs. the bulbar cohorts, respectively), as well as how an older age at onset corresponds to a more rapid progression to death. When compared, the PRO-ACT and the ALS–BS patient populations present some similarities in terms of natural progression of the disease, as well as some differences in terms of observed trajectories plausibly due to the trial scheduling and recruitment criteria.

**Conclusions:**

We exploited PM to provide an overview of the evolution scenarios of an ALS trial population and to preliminary compare it to the progression observed in a clinical cohort. Future work will focus on further improving the understanding of the disease progression mechanisms, by including additional real-world subjects as well as by extending the set of events considered in the impairment trajectories.

## Background

Amyotrophic Lateral Sclerosis (ALS) is a rare neurological disease primarily affecting motor neurons, causing progressive paralysis of voluntary muscles and leading to death following respiratory insufficiency, usually within 3–5 years from onset. Clinical picture is characterized by signs of both upper and lower motor neuron involvement combined in a variable manner in terms of severity and distribution, which results in a consistent clinical heterogeneity. Strong differences in rate of progression, age of onset, site of onset (bulbar vs. spinal), duration of the disease, and association with other conditions are frequently observed in clinical practice and contribute to the wide clinical heterogeneity, making diagnosis as well as prognosis, clinical trial design, and development of therapies very challenging [1]. Genetic heterogeneity is also well known in ALS with more than 40 identified genes playing a causal role or conferring susceptibility, thus further contributing to the biological heterogeneity of the disease [2].

In this context, there is a significant need for data-driven tools capable of modeling the progression of ALS to describe the manifold nature of this disease, to identify risk factors, to group patients based on similar evolution patterns, and to support personalized prediction. For this reason, there is a growing interest in methods for mining and analyzing the progression of ALS, particularly by considering longitudinal clinical data.

### Related work

In the last decade, a number of predictive tools have been developed to forecast ALS clinical end-points such as the rate of future progression, the impairment of functional capabilities, and survival, the latter often coded as a composite outcome including the administration of tracheostomy. Moreover, several data-driven and clinical criteria aimed at stratifying patients into meaningful subgroups based on their clinical characteristics or prognosis similarity have been proposed.

In 2012 and 2015, two distinct DREAM (Dialogue for Reverse Engineering Assessments and Methods) Challenges have been organized with the purpose of boosting research on ALS towards the development of computational tools for prediction and stratification purposes, respectively [3, 4]. Starting from the data of ALS patients included in both clinical trials and real-world registries and collected over the 3 first months of visit, the participants were asked in a case to predict the progression of the disease and the survival in the subsequent months, in the other to identify and characterize clinically relevant sub-populations of patients. Various solutions were proposed, with Bayesian Trees, Random Forests (RF), and non-parametric regression being predominant in the prediction challenge, and with RF, Gradient Boosting Regression Trees, Support Vector Machines, and Gaussian Process Regression largely used for stratification. As a result, these initiatives have successfully led to the identification of features with a significant predictive potential and/or discriminative effect on the patient clusters, shedding a light on the phenotypic heterogeneity of ALS. It is interesting to observe how, in both challenges, participants were provided with data collected over a time window that could be used to model the progression trend and, consequently, to infer the subjects’ prognosis or their sub-population membership. Participants implemented different strategies to effectively extract information from the supplied dynamic features and use it in their models: in the prediction challenge, for instance, some teams converted the time-resolved data collected in the first 3 months of visit of each patient into a set of derived static features representing their evolution, such as the minimum, maximum, or slope of their values.

This embedding and, more in general, the use of longitudinal information is in contrast to the choice made in other state-of-the-art works, where the prediction/stratification task is addressed starting from the patients’ information collected at a static point only, like for instance their first visit (e.g., [5–7] for stratification, [5, 8] for prediction).

As another option to effectively use the dynamically collected patient information for describing the patient status and producing a prognostic prediction, Carreiro et al. proposed an approach based on patient snapshots and time windows [9]. In this work, the patient condition at a given time point is enriched by grouping together the clinical tests performed inside a considered time interval using hierarchical clustering. These snapshots are then used to predict the probability of each patient to require assisted ventilation after a given time window from the clinical evaluation, using state-of-the-art classifiers. The authors showed how combining the use of patient snapshots and time windows significantly improved the performance with respect to a baseline Cox proportional hazards regression model which supported only the use of temporal windows in the analysis. This snapshot-based approach has also recently been used by Müller et al. [10]: using longitudinal data from a large cohort of ALS patients treated in a Portuguese ALS clinic in Lisbon, the authors employed recurrent neural networks to predict the decline in breathing capability, as measured by clinical administration of non-invasive ventilation (NIV). Noticeably, in this work a specific focus was also put on the explainability of the learned predictive models, using Deep Shapley Additive Explanations (Deep-SHAP) [11] and promoting the communication of the outcomes with clinical researchers to assess the approach practical utility in aiding prognostic prediction and improving patient care.

The whole range of temporal information included in six real-world clinical registers has been recently used to build two distinct disease progression models based on Dynamic Bayesian Networks [12]. Such models are able to catch and explicitly represent the relationships occurring among the variables constituting the datasets over time in terms of conditional probabilities, as well as the pathways along which they influence the disease progression. Therefore, the networks can be employed to both identify inter-dependencies of interest and to simulate the prognosis of new sets of patients starting from their information collected during the first visit. Moreover, the implemented tool allows to predict the time to loss of independence in four characteristic functional domains affected by ALS and survival, as well as to assess the effect of different biomarkers on the disease course.

With the aim of better explaining the progressive nature of ALS and exploiting it for improving personalized prognostic forecasting, Carreiro et al. employed a temporal mining-based approach, namely the Sequential Pattern Mining (SPM), for finding the frequent progression patterns within an ALS population [13]. A pattern consists of a series of transactions, each of them being an evaluation performed during the patient’s follow-up and constituted by items coded as a pair (exam, value). Patterns allow to characterize the progression behaviors in the population, but, as a limitation, require the data variables to be prior categorized. The most frequent patterns were then extracted and included as features in a classification setting for forecasting the need of NIV by the patient, providing an enhancement in the classification performance. Similarly, Martins et al. employed a combination of Itemset Mining [14] and SPM to discover both disease presentation patterns and disease progression patterns, from static data collected at diagnosis and temporal data from patient follow-up, respectively [15]. These patterns were then used as features in prognostic models, allowing predictions to take disease development into account and improving model interpretability.

In other works, patient disease trajectories were modeled in terms of variations of clinical indices collected in the clinical practice to assess and keep track of the patient status. For instance, Gomeni et al. modeled ALS progression in terms of individual patterns of decline of functional abilities [16], as measured through the revised Amyotrophic Lateral Sclerosis Functional Rating Scale (revised ALSFRS, or ALSFRS-R) [17]. Ackrivo et al. employed Forced Vital Capacity (FVC) trajectories to identify three clinical phenotypes of ALS respiratory progression [18]; Thakore et al. gained insights into progression of ALS by applying Markov models to ALS stages coded by multiple functional scores, being able to adequately describe transitions from a progression stage to the next, especially in the first phase of the disease [19].

The dynamic nature of ALS as recorded in the clinical records can also be exploited for other than descriptive or predictive purposes, such as for imputing missing data. Tavazzi et al. recently proposed a strategy based on a mutual information-weighted k-nearest neighbors (k-NN) algorithm able to impute clinical datasets constituted by mixed-type static and dynamic variables [20]. By employing as k-NN samples windowed sets of visits properly structured and an ad hoc-developed similarity metrics able to compare the disease evolution while handling the simultaneous presence of missing information in different types of variables, the method was proved to effectively impute an ALS clinical register.

### Aim of this work

Despite the growing interest in understanding and predicting the progressive nature of ALS, hardly any of the aforementioned works allows and the same time both to neatly represent the entire evolution of the disease and to effectively describe how the patients’ characteristics affect the progression’s trajectory. Moreover, the given models cannot provide an easy to understand and comprehensive overview of the most probable scenarios of evolution of the patient status, rather often limiting the forecasting to a single progression end-point or assigning the subject to a single, static progression phenotype.

In this work, we aim at modeling the progression trajectories of ALS employing an alternative approach based on Process Mining (PM). PM is a relatively young discipline arose in the process management context, that provides methodologies to support the analysis of operational processes starting from the Event Logs (ELs) recorded by an information system [21]. Specifically, an EL is a collection of relevant activities (or events), each referred to a case and labeled with its occurrence time, plus an optional set of features, defined attributes, that characterize the case or the activity. Namely, the list of events of each case constitutes a *trace* in the EL.

There are three categories of process mining techniques:

1. *Process discovery*, which aims at mining the data to represent the process that produced them [22];
2. *Conformance checking*, which allows to assess the compliance of an EL with regards to a given process model or, vice versa, to what extent a given process model reflects an input EL [23];
3. *Process enhancement*, which enables to improve the efficiency of an existing process model using process data, by providing means for problem diagnosis or delay prediction, as well as recommending process redesigns and supporting decision making [24].

PM has recently gained momentum in a variety of domains, including healthcare. As more digitalized data become available, more processes can be mined, providing insights on patient care and resource allocation. Specifically, the processes in this context can be distinguished into clinical and administrative/organizational [25]. As reported in two recent surveys of PM in healthcare [26, 27], clinical processes are the object of most of the state-of-the-art works, with applications including Cardiology (see, for instance, [28, 29]), Oncology (e.g., [30–32]), Primary Care (e.g., [33, 34]), and Emergency Care (e.g., [35, 36]). On the other side, organizational processes applications comprehend billing [37, 38] and reimbursement [39] processes.

As a limitation, most of the PM-based analyses performed in the healthcare domain focus on the application of classical Process Discovery methods (such as the Heuristic Miner [40] and the Inductive Miner [41]), missing the opportunity to support the discovered processes with the adoption of statistical inference. With specific reference to Process Discovery, this is reflected in two main drawbacks: (i) the evidence of the PM results is not systematically supported by statistical inference, and (ii) PM does not exploit the approaches that are most familiar to medical doctors, hence reducing the trust in the results and analysis.

To address the above-mentioned drawbacks of traditional PM-based analysis, in this work we implement a novel process-oriented analysis for mining and describing the progression of ALS, gaining insights on its mechanisms and performing inferential studies. Specifically, we focus on the progressive impairment of the patients’ functional abilities as the disease progresses, employing the ALS Milano–Torino staging system [42] to quantitatively characterize the health worsening over time, together with the survival outcome.

Starting from two distinct ALS datasets, namely, a clinical trial and a real-world one, we first structure the patients’ longitudinal information collected during their follow-up as an EL. Then, by employing Process Discovery techniques such as the Directly-Follows Graph and the CareFlow Miner [31], we discover the underlying processes that generated the patients’ ELs. We are then in the position to analyze the patterns of evolution over the populations, and to investigate the inferential potential of the mined processes in terms of capability to describe and differentiate the prognosis—for instance, in terms of timing or sequence of events—based on the value of specific covariates at baseline. Finally, we compare the process built on the clinical trial data with the one obtained on the real-world setting data, to assess the differences and similarities among the pathways followed by two populations, that have been recruited and followed-up according to different criteria and scheduling. To the best of our knowledge, this is the first time that such a principled PM-based analysis has been performed on ALS: it is a step that can foster and support a better understanding of the hidden processes behind the progression of this disease.

## Methods

The proposed methodology includes the following three main phases:

1. *Data structuring*: the data are processed and shaped in the form of an EL, with attributes and events formatted according to the aims of the analyses. More ELs can be produced, at this point, to highlight different aspects of the data. The ELs can be iteratively refined by removing irrelevant, incomplete or wrong information to enable the next analysis stage. Specifically, here we derive for each patient a trace consisting of tuples < patient ID, Event, Timestamp, *Attributes*>, with the Events being the functional impairments and the survival status (death/censoring), and the *Attributes* being a small set of features characterizing the patient baseline condition.
2. *Process discovery*: this phase aims at discovering the processes that generated the data, starting from the provided EL. However, noisy data may compromise the reliability and the accuracy of the mined processes. A number of techniques, like the Alpha algorithm [22], the Heuristic Miner [40], the Fuzzy Miner [43], or the Inductive Miner [41] can be employed in this phase. As a positive feature, many PM algorithms provide a graphical output of the mined processes which facilitates both their interpretation and communication. In this work, we exploit two state-of-the-art Process Discovery techniques, namely the Directly-Follows Graph and the CareFlow Miner [31].
3. *Process analysis*: both descriptive and inferential analyses can be performed on the mined processes, allowing the implementation of the classical steps of statistical analysis, here in a process-oriented setting [32].
  From a descriptive point of view, processes can be investigated to identify volumes, general data distribution and reveal statistical biases or the presence of bottlenecks. Events can be inspected in terms of cross-relationships, causality, or position in the pathways. Moreover, an analysis of the mined trajectories can help in identifying the most recurrent patterns as well as in first outlining how the data may deviate from the expected behaviors.
  From an inferential perspective, processes can be analyzed to highlight the events or attributes that characterize specific behaviors in the sample population. For instance, cases can be stratified according to their features and compared in terms of pathways or outcomes, thus estimating the impact of different data characteristics and possibly revealing markers of evolution. This kind of analysis allows to reveal statistical dependencies and correlations, with indicators such as *p*-values or confidence intervals, among data. This is a pivotal step of the pipeline, that allows to either confirm or reject formulated hypotheses, and to support the formulation of further theses as well. Similarly, a process built on a reference EL can be compared with the one resulting on an independent EL—here used as a kind of external testing set—to confirm or highlight differences in terms of trajectories, event frequency, or timing. If the descriptive and/or inferential evidences are support, domain experts can help to generate communicative reports, also integrating the graphical representation of the processes often provided as algorithms’ output. This can be used as the basis for the implementation of Decision Support Systems (DSSs), that can improve the decision process for subsequent patients.

We performed the analysis using pMineR [44], a software library in R specifically born to support PM analysis in healthcare and recently improved in the direction of exploring differences among the mined pathways.

### Data and preprocessing

In this work, we employed data of ALS patients sourced from two distinct datasets, that is, a clinical trial data collection and a real-world clinical register. This choice allows to assess differences and similarities between the processes happening in two distinct settings.

#### The PRO-ACT dataset

As a first data source, we considered the Pooled Resource Open-Access ALS Clinical Trials (PRO-ACT) dataset [45], that is the largest publicly available repository of merged ALS clinical trials data. This dataset includes demographic and longitudinal clinical information collected over 10 million data points for more than 10,000 fully de-identified patients who participated in industry, foundation, and academia sponsored clinical trials. PRO-ACT includes data from both placebo and pharmacologically treated patients. According to the provided documentation, the medications tested in the trials were found to be no better than placebo with respect to their effects on ALS progression. For each patient, PRO-ACT collects a number of static features tracking the patients’ personal information, family and medical history, and data characterizing the first phase of the patient disease (e.g., the site of onset). Then, for each dynamic feature collected during the trial follow up —like vital signs, results of lab test, or the assessment of functional scores tracking the patient status—the value of the specific examination together with the temporal distance from the onset is reported.

On the one hand, PRO-ACT represents a precious resource for research studies, thanks to its large sample size and the high frequency of its visits that allow a precise characterization of how ALS progresses in the study population. On the other side, it consists of data from a selected pool of patients monitored over a limited period, thus not fully representing the general ALS population and possibly limiting the generalizability of the findings [46].

Starting from the comma-separated values (CSV) files downloadable on the PRO-ACT website,^1^ we preprocessed the data as follows. First, we removed the patients with an unrecorded onset date or reporting an onset following the trial start. Then, to better characterize the progression of the disease, we filtered out the subjects without functional assessment in any of their visits or without at least one visit within 6 months of the study begin. Then, we removed the visits without a functional assessment or performed before the trial start. This preprocessing reduced the number of subjects from 10,723 to 5,389, for a total of 68,654 visits performed during the trials. Table 1 reports a characterization of the preprocessed PRO-ACT dataset.

Then, for each visit we converted the functional scoring system available in the data, that is, the ALSFRS score [47], into the Milano–Torino staging (MiToS) system [42]. While the ALSFRS evaluates the patient functional status through a 10-item questionnaire referred to different daily and vital abilities, each rated on a 0–4 point scale, the MiToS system defines 4 functional domains (namely Walking/Self-care, Swallowing, Communicating, and Breathing), each coded as a binary variable that switches from 0 to 1 when the specific domain is impaired. By effectively characterizing the evolution of the disease over time, the use of the MiToS also allows to overcome the limitations of the ALSFRS, mainly consisting in a reduced ability of catching the worsening in the advanced phase of the disease [48, 49] and the absence of agreement on a threshold at which changes in the ALSFRS scores are viewed as an important transition point in functional status [42].

After obtaining the MiToS scores, we aggregated them into a new feature that encodes the overall status of the impairment. Specifically, we coded for each visit a string *M_abcd* consisting of four elements $\{a, b, c, d\}$, each corresponding to a functional domain in the following order: (a) Walking/Self-care, (b) Swallowing, (c) Communicating, and (d) Breathing, and assuming a value equal to 1 if the domain was impaired and 0 otherwise. For instance, M_0000 corresponds to no recorded impairments at that visit, while M_0011 to a recorded impairment in both Communicating and Breathing.

Finally, we converted the dataset into an EL, considering as activities: the *disease onset*, the *trial start*, the status of the *MiToS impairments* coded as above, and the *death* or the *censoring* event. We made the choice of coding the survival information in this way in order to be able to represent the censoring event in a dedicated region of the PM graphs (precisely, the “Censored” status itself) and be thus able to discern the contribution of complete versus truncated observations on the trajectories. This resulted in an EL of 26,426 logs referred to the 5,389 included subjects. For each subject trace, we coded as attributes some static information, namely, the age at onset and the site of onset, to perform stratification analyses for assessing their effect on the disease trajectories.

**Table 1:** Demographic and clinical characteristics of the ALS populations included in the study

Features		PRO-ACT	ALS–BS	*p*-value
*n*		5389	43	–
Sex	Female	2018 (37.4)	21 (48.8)	0.123
	Male	3371 (62.6)	22 (51.2)	
Onset site	Bulbar	1122 (20.8)	11 (25.6)	<0.01 *
	Spinal	3875 (71.9)	31 (72.1)	
	Spinal and bulbar	59 (1.1)	1 (2.3)	
	Other	333 (6.2)	0 (0.0)	
Age at onset	[years]	55.26 ± 11.68	59.14 ± 10.84	0.034
FVC at baseline	%	82.24 ± 17.94	96.47 ± 15.35	<0.01
Weight at baseline	[kg]	75.86 ± 15.79	68.85 ± 9.20	0.019
Total ALSFRS at baseline	[score/40]	30.12 ± 5.47	36.12 ± 9.20	<0.01
Disease duration	[days]	1063.03 ± 456.22	1241.67 ± 722.13	0.201
Follow-up duration	[days]	407.06 ± 175.27	931.49 ± 627.49	<0.01
Follow-up visits		12.74 ± 6.23	7.44 ± 2.80	<0.01
Survival	Censored	3967 (73.6)	23 (53.5)	<0.01
	Dead/Tracheostomized	1442 (26.4)	20 (46.5)	

Numerical features are described using mean ± standard deviation, categorical features are described using cardinality (percentage). Kruskal-Wallis and $\chi ^{2}$ tests at .01 significance level were used for assessing the equality of the distributions of the continuous and categorical variables, respectively, in the two datasets. * indicates a non-complete fulfillment of the statistical test’s assumptions. FVC = Forced Vital Capacity, ALSFRS = ALS Functional Rating Scale

#### The ALS–BS dataset

In order to extend the investigation of the ALS patterns of evolution on real-world patients, we included in this work also a second data collection (hereinafter ALS–BS) consisting of patients followed up in a tertiary clinical context. Specifically, we employed the data collected during routine clinical assessment at the NeMO - NEuroMuscolar Omnicenter of Brescia and at the Neurology Department of the Spedali Civili of Brescia (Italy), two interconnected highly specialized clinical centers dedicated to diagnosis, treatment and research in the field of neuromuscular diseases. This dataset collects in an anonymized way the salient clinical data of 43 ALS patients classified on the basis of El Escorial-Awaji criteria [50] and diagnosed between between January 15th, 2014 and December 1st, 2020, including age and site of onset, vital signs, distribution of muscle weakness and atrophy, neurophysiological studies, functional scores, and genetic results. Table 1 reports the characterization of the patients included in the ALS–BS, together with a comparison of the distribution of the features in the PRO-ACT versus the ALS–BS dataset performed in terms of Kruskal-Wallis and $\chi ^2$ tests at .01 significance level for the continuous and categorical variables, respectively.

We preprocessed the ALS–BS data in the same way of PRO-ACT (with the exception, given the different nature of the dataset, of the trial start event), obtaining an EL of 43 traces for a total of 236 events.

### Process discovery methods

In this work, we employed two distinct Process Discovery methods, namely the Directly-Follows Graph (DFG) and the CareFlow Miner (CFM), to model the evolution of ALS in terms of progressive impairment of the MiToS functional domains and survival. As per their implementation in pMineR, both the DFG and CFM return the mined processes under a graphical form, directly providing the user with descriptive statistics such as the cardinality of the subjects following the different paths or the probability of transition between events. Moreover, they allow the comparison of processes built on different datasets, highlighting differences and similarities.

First, we performed a set of analyses using the DFG on the PRO-ACT dataset only, considering the entire population or sub-cohorts stratified by their site of onset. Then, CFM is discovered on the PRO-ACT dataset to reveal the most frequent path, time and statistical differences in sub-cohorts split on the base of the age of the patients and the onset site. Finally, we compared the DFG process obtained on PRO-ACT with the one built on the ALS–BS dataset, focusing on the variations in the transition probabilities between consecutive events and further inspecting the functional worsening transition times in the two datasets.

In the remaining part of this section, we detail the two above-mentioned techniques.

#### Directly-Follows Graph

The DFG is probably one of the most intuitive graphical languages which simply connects two nodes representing two events with an edge when they are subsequent in at least one trace. Despite its simplicity, it can provide useful insights into the actual processes. To prune the graph, in order to make it easier to analyze, different thresholds can be applied (e.g., based on the absolute/relative number of transitions, timing, etc). In pMineR, the DFG is available by the class *firstOrderMarkovModel* implementing a number of features to allow the exploration of:

- *time to fly:* Given a pair of consecutive or non-consecutive events, the time to fly corresponds to a kernel density estimation function of the times needed to move from the starting to the destination node computed over all the population;
- *survival functions:* Kaplan–Meier (KM) curves can be built, including possible constraints to select the cohort(s) of interest (e.g., passing or not through specific nodes, nodes playing the role of *censoring*), and then compared with a log-rank test to check statistically significant differences;
- *deltaGraphs:* Two DFG graphs, built for instance on two cohorts with different baseline characteristics or followed up in distinct clinical centers, can be overlaid to measure the differences in terms of transaction probabilities between nodes. Thresholds can be applied to reduce the noise and focus only on the most relevant differences.

#### CareFlow Miner

The CFM algorithm implemented in pMineR is an extension of the original version presented in [31]. Starting from a *root* node, each trace in the EL contributes to create a branch of a tree where the top level (i.e., the first after the root) represents the first event of each trace, and the next levels sequentially correspond to further events of each trace. In the resulting CFM graph, each node is labeled with the name of the corresponding event and additional information such as the number of patients passing through that node or statistics about the time needed to reach it.

On the one hand, on complex ELs the tree tends to explode in terms of nodes and edges. To avoid the so-called *Spaghetti Effect*, a CFM tree is normally pruned on the base of a threshold, to exclude highly infrequent paths and reduce its complexity. On the other hand, the meaning of the language is easy to understand and the algorithm is clear. This feature, differently from existing PM algorithms such as Alpha Algorithm or Fuzzy Miner, helps in reducing the psychological barrier the clinician may have with respect to what they can feel as *black box* solutions [24].

By itself, CFM is an algorithm able to show the most frequent paths, thus revealing outliers and suggest further investigations, but it is not able to provide confidence intervals on their occurrence or *p*-values on the effect of specific data features on the followed trajectories. pMineR overcomes this limitation by offering the opportunity to compare two CFM graphs corresponding, for instance, to two different cohorts of patients, such as male versus female, with versus without a morbidity at baseline, as so on. The two obtained CFMs are measured node by node with a Fisher’s exact test or a $\chi ^2$ test (depending on the cardinality of patients passing through the node) for dichotomic categorical variables, or with a Wilcoxon–Mann–Whitney test for the continuous one, to compare the time needed to move from the root to the node or to move from the node to a given possible event (e.g., death).

#### Patient trajectories comparison between datasets

To measure the difference between the trajectories of progression in PRO-ACT versus ALS–BS, we leveraged on the simplicity and intuitiveness of DFG, and compared the processes mined on the two datasets by building a *deltaGraphs* on the two ELs. Then, we computed the kernel density estimation of the time-to-fly between each couple of consecutive and non-consecutive events for both the datasets. Finally, for each common transition, we compared the different time of transitions with a Mann-Whitney test for that cases where the cardinality of transitioning subjects was high enough; when the number of available samples was lower than 5, we employed a qualitative visual inspection of the densities instead. Where the cardinality was high enough, we also inspected the difference in terms of median transition times.

## Results and discussion

This section presents the results of the performed analysis focusing on the data characteristics and the discovered processes. Based on the number of available patients and events, we focused our main analyses on the PRO-ACT dataset. We then considered the ALS–BS for comparison purposes, with the aim of identifying the differences in terms of impairment trajectories in a, albeit limited, real-world dataset.

### Data characterization

Table 1 reports a characterization of the two cohorts of patients included in this work. Although reduced in dimensionality, the ALS–BS dataset (sample size n = 43) shows significant similarities to PRO-ACT in terms of patients’ sex (p=0.123), age at onset (p=0.034), weight at baseline (p=0.019), and disease duration (p=0.201). In contrast, FVC and total ALSFRS as measured at the first visit significantly differ between the two datasets, possibly as a consequence of the recruitment criteria for the trials. For what concerns the site of onset, we can notice that PRO-ACT presents a further level with respect to ALS–BS, namely “Other”. It was however not possible to better investigate its meaning starting from the dataset documentation. We can notice how in PRO-ACT the observed follow-up is shorter (mean ± SD equal to 407.06 ± 175.27 days in PRO-ACT vs. 931.49 ± 627.49 days in ALS–BS), and the number of censored patients is higher (73.6% vs. 53.5% in PRO-ACT and ALS–BS respectively), coherently with a limited duration of trials. Although the almost halved follow-up, it is worth noticing how the average number of visits in PRO-ACT is approximately doubled with respect to ALS–BS. The improved visit frequency characterizing the trial follow-up has a direct repercussion on the granularity at which the MiToS impairments are collected and, correspondingly, at which a possible worsening is tracked. In line with expectations [46], these numbers actually reflect the presence of meaningful differences between trial and real-world populations, especially in terms of recruitment criteria and observational scheduling.

### Process discovery on PRO-ACT

Here we report and comment the processes built on the PRO-ACT dataset and the investigation of the effect of baseline covariates in discriminating the patients’ trajectories.

#### Process discovery based on DFG

**Fig. 1:**
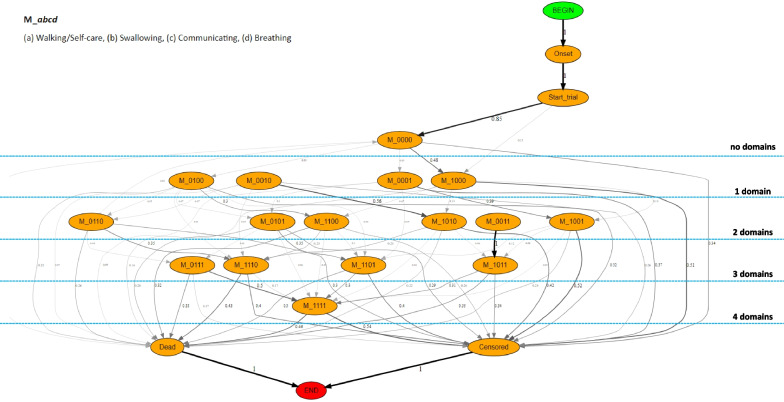
DFG graph representing the paths followed by the study population, delineating the increase in disability experienced by the subjects. Only the arcs with a transition probability $>0.03$ are shown

Figure 1 shows the process obtained mining the whole PRO-ACT population with DFG. For the sake of readability, we report only the edges with a transition probability > 0.03 with respect to the cardinality of the patients in the node where the edge origins. Notably, the topological organization of the obtained DFG reflects the increasing trend of functional domains affected as ALS progresses: none at the top (corresponding to the first visit right after the trial start for 85% of the patients), one domain thereafter, and so on until a final state among M_1111 (all domains affected), censored, or death is reached.

**Fig. 2:**
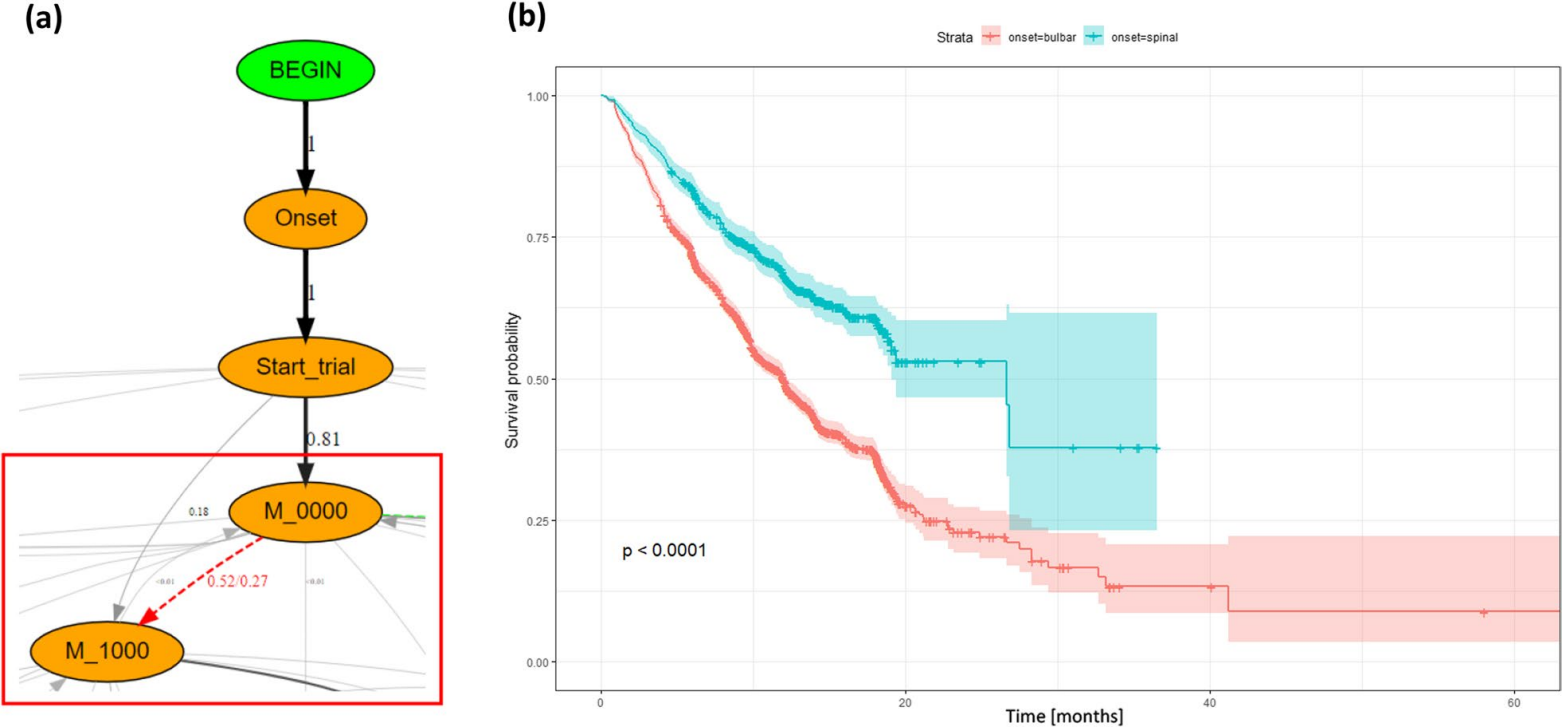
**a** Zoom on the DFG *deltaGraph* obtained stratifying the population by onset site (spinal vs. bulbar). The highlighted edges represent an increased transition probability for the spinal (red) or bulbar (green) cohort, thresholded for displaying only differences between the probabilities greater than 0.2. **b** KM curves of the time passing from M_0000 (no impaired domains) to M_1000 (Walking/Self-care domain impaired) for the two cohorts. The log-rank test shows statistically significant differences between the cohorts. $+$ indicates censored subjects

We then employed DFG to shed some light into the kinetics of different clinical sub-types of ALS patients. After dividing the PRO-ACT population into two distinct groups based on their main sites of onset (spinal vs. bulbar), we compared their trajectories by mean of a DFG *deltaGraph*, under the hypothesis that their impairment patterns would differ. Specifically, we expected the spinal patients to manifest an early impairment in the Walking/Self-care ability, and the bulbar to have an early deterioration of the Communication and/or Swallowing functionalities. A zoomed section of the resulting DFG *deltaGraphs* is reported in Fig. 2a. In order to highlight the main differences of the two cohorts, we thresholded the graph for displaying only transition differences > 0.2. We can confirm our hypothesis by observing, for instance, that the transition probability from M_0000  (first visit without an impairment) to M_1000 (impairment in Walking/Self-care) results enhanced for patients with spinal onset, namely with 52% of them losing mobility as first domain versus 27% of the bulbar ones. The corresponding difference in terms of transition times can be inspected by analyzing the KM curves reported in Fig. 2b, which proves to be statistically different (log-rank test p < 0.0001). When computing the KM curves, we included the possibility that some patients were censored before reaching the ending state of interest (here M_1000). These cases are represented in the plot with a $+$ symbol. It is worth noticing how, in this kind of analysis, it is of course pivotal to take into account the cardinality of the subjects following the considered edges in order to avoid a misinterpretation of the results due to possible overfitting of a very specific class of cases.

#### Process discovery based on CFM

**Fig. 3:**
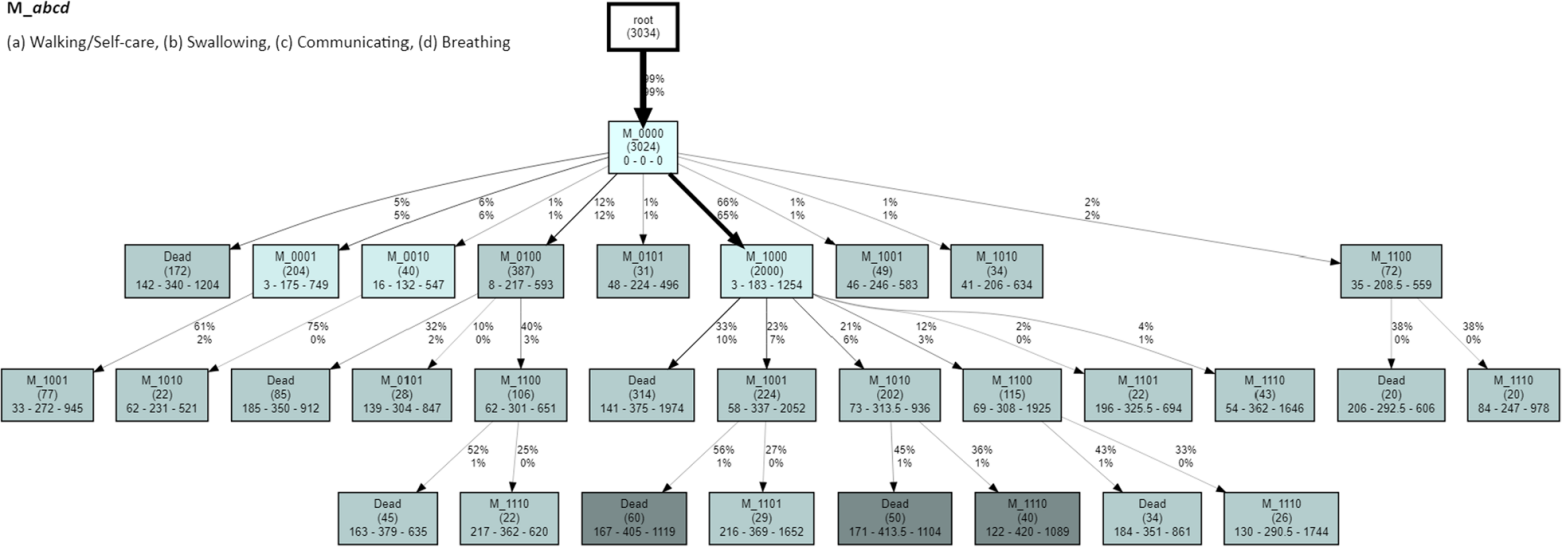
CFM graph built starting from M_0000. Each node reports the total number of patients passing through it (round brackets) and the min-median-max time, in days, needed to reach it from the root. Colors are graded on the median times, with intervals: <100, 101-200, 201-400, and >401 days. The edges report the percentage of patients passing through the child node with respect to the previous node (above) and the entire population (below). The graph has been thresholded for displaying only the pathways transitioned by at least 10 subjects

Figure 3 shows the most frequent patterns mined through the CFM, here built starting from the node M_0000. The graph has been thresholded for displaying only the pathways transitioned by at least 10 subjects. For each node, the total number of patients passing through is reported in brackets, while the minimum, median, and maximum time (in days) needed to reach that node from the root is shown on the second line. The edges report the percentage of patients passing through the child node with respect to the previous node (above) and the entire population (below). It is worth noting that these percentages may not sum to 1 in the graph as a result of the out-filtered infrequent patterns.

We can observe how, in the process mined using CFM, we can rediscover the DFG transitions with higher cardinality. It is the case, for instance, of the edge from M_0000 to M_1000, that emerges as predominant in both processes. It is worth reminding that by design the CFM potentially multiplies the number of times that a same node appears in the graph. This happens when the same event happens in distinct paths and is due to the fact that while the DFG only represents the transition between an Event and the next one (with no memory of the past), the CFM keeps the memory of all the steps happened before. For this reason, the former can be a cyclic graph while the latter is necessarily acyclic.

As the DFG, the CFM tree can also be stratified by a variable of interest in order to assess any significant differences in the paths’ occurrence according to the subjects characteristics. Guided by clinical hypotheses, we explored the distribution of the subjects in the nodes with respect to their baseline or static covariates, focusing in detail on:

- age at onset (quantized into two levels according to its mean value = 55 years, see Table 1) on the death, testing that older age at onset corresponds to a worse outcome, as reported in [51];
- onset site (spinal vs. bulbar) on the occurrence of the impairments, further checking that a spinal onset early affects motor skills while a bulbar one causes early dyspnea, dysphagia, or dysphonia.

**Fig. 4:**
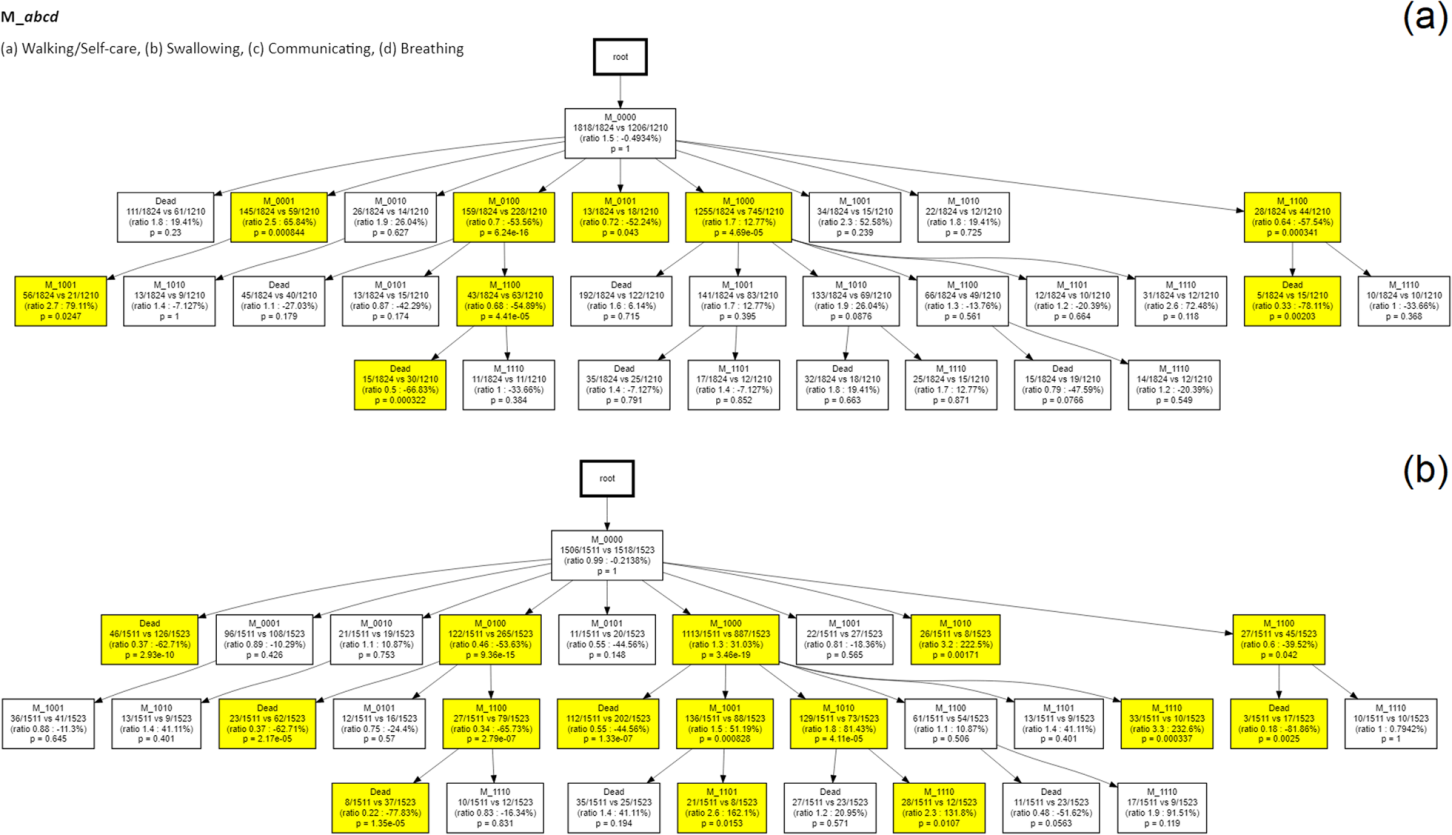
CFM graphs built starting from M_0000 and stratified for **a** quantized age at onset or **b** onset site. Each node reports the number of patients passing trough it for each cohort (young/aged onset and spinal/bulbar onset, respectively). Moreover, the ratio of the two cardinalities with respect to the initial populations is reported in the round brackets, followed by the *p*-value for the Fisher’s exact/$\chi ^2$ test. The node box is colored in yellow if the *p*-value is lower than a given threshold (here 0.05). Both the graphs have been thresholded for displaying only the pathways transitioned by at least 10 subjects

Figure 4 shows the obtained graphs. Each node reports the number of subjects passing through it for each cohort (young/aged onset or spinal/bulbar subjects, respectively), with the ratio in brackets, and the p-value of the Fisher’s exact/$\chi ^2$ test, depending on the cardinalities involved in each node, on their distribution.

Figure 4a reports the graph obtained by stratifying the population by the age at onset. We can observe how the aged patients actually experience an early death, even if only on specific trajectories, namely: (i) M_0000 $\rightarrow$ M_0100 $\rightarrow$ M_1100 $\rightarrow$ Dead, with ratio young/aged equal to 0.5, and (ii) M_0000 $\rightarrow$ M_1100 $\rightarrow$ Dead, with ratio young/aged equal to 0.33. Figure 4b refers to the stratification based on the onset site. Here, it emerges a significant predominance of a first impairment in the Walking/Self-care domain for the subjects with spinal onset (M_1000, ratio spinal/bulbar equal to 1.3), and a significant predominance of first impairment in the Swallowing domain for the bulbar onset subjects (M_0100 with a ratio spinal/bulbar equal to 0.46).

These results partially match with the expectation, with the effect of the stratifying variables being investigated not emerging over all the trajectories due to a limited subject cardinality along some branches.

### Trajectories comparison of PRO-ACT and ALS–BS based on DFG

**Fig. 5:**
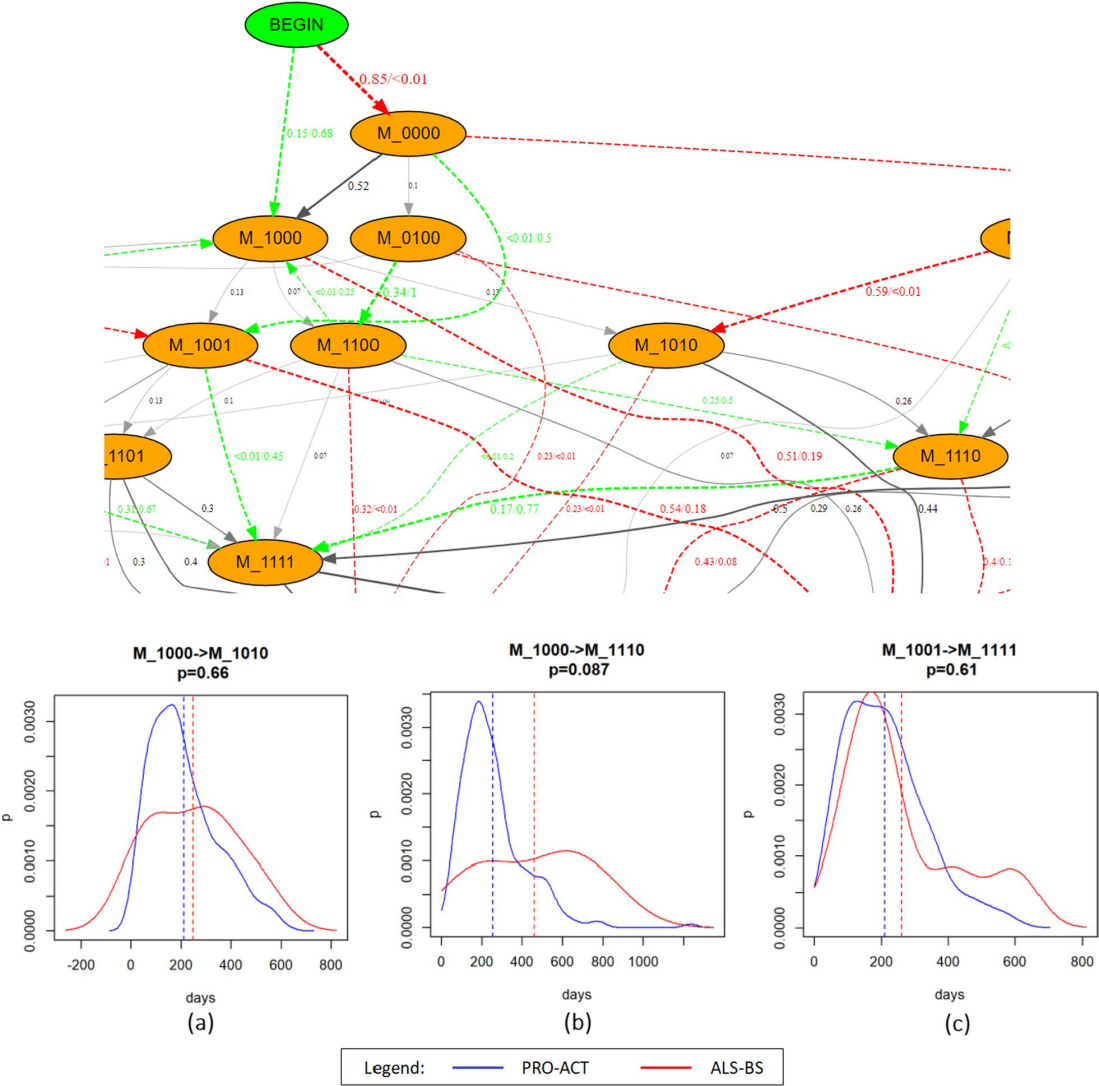
On the top, a portion of the DFG showing the differences between PRO-ACT and ALS–BS in terms of probability to move from a state to the next one is shown. The gray edges represents the transitions whose probabilities differ for less than 0.2 in the two datasets. When this gap is larger, the edge is green if ALS–BS has a probability higher than PRO-ACT, red otherwise. On the bottom, the kernel density estimation of the probability to move (**a**) from M_1000 to M_0101; (**b**) from M_1000 to M_1110, and (**c**) from M_1001 to M_1111 are reported, with PRO-ACT in blue and ALS–BS in red, and with the vertical dotted line indicating the median of the distributions

A comparison of the two datasets through the DFG is shown in Fig. 5, where the differences in terms of transition probability from an event to a consecutive one are shown with different colors (red or green edges) if differing for more than 0.2 between the two datasets. In contrast, gray edges indicate transitions with similar probability. Even if this analysis is limited to adjacent events, and can thus not be exploited to evaluate long-time transitions (which might have additional events in between), it turns out to be particularly effective in revealing errors in the data (e.g., an improbable regression of the disease consisting of the recovery of a previously impaired domain). In our case, the large number of observable differences between the two datasets is probably mainly due to the different recruitment/dropping criteria between PRO-ACT and ALS–BS: in the former, the protocols were more standardized and the recruitment more homogeneous (even if with a higher percentage of early-censored patients), in the latter the patients might on one side have had the first contact with the clinical centers at various stages of the disease and, on the other, have been more frequently assisted until the final phases of the disease. It also has to be mentioned that the reduced cardinality of the ALS–BS dataset might constitute a limitation to the observed behaviours. The bottom part of Fig. 5 shows an example of comparison between the two datasets of the kernel density estimation for the common transitions: (a) from M_1000 to M_1010, (b) from M_1000 to M_1110, and (c) from M_1001 to M_1111. Notably, (b) and (c) do not correspond to specific edges in the DFG, representing instead the time needed to move from the source state to the target state in an arbitrary number of steps.

By comparing the median times of the distributions, we can observe how, in our examples, the ALS–BS red dotted line is always situated on the right side of the PRO-ACT dotted blue line. In other words, the ALS–BS population has in all these cases a longer median time to event. It is worth noticing how for the consequent events, whose representation is included in the DFG, the analyses of the transition probabilities and of the density distributions can be paired. For the case reported in Fig. 5a, for instance, we can conclude that the transition from the only Walking/Self-care impairment (M_1000) to the condition where also the Communication is damaged (M_1010) is similar in the two datasets both in terms of transition probability (gray edge in the DFG) and of median time of transition (dotted lines in plot (a)), while the corresponding time distribution slightly differ in terms of kurtosis.

## Conclusions

In this work, we implemented a process-oriented investigation of the disease trajectories of ALS modeled as a succession of impaired functional domains. Starting from two distinct ALS datasets, namely a clinical trial and a real-world one, and after a preprocessing phase aimed at cleaning data and extracting the events of interest in the form of an EL, we mined the processes that generated the observations by considering two Process Discovery algorithms, namely DFG and CFM. Enriched in their original formulation, we employed these algorithms to also reveal how the pathways may differ based on patient static attributes. We studied the processes in terms of trajectories, probabilities, and times of transition between both consecutive and non-consecutive events, and tested clinical hypotheses to better explore the process characteristics linked to nature of the datasets and the impact of the patients’ characteristics at baseline on their progression.

To the best of our knowledge, this work represents the first attempt of modeling ALS trajectories using a PM-based approach, and one of the few applications of these techniques in the domain of neurodegenerative diseases. With respect to existing methodologies that focus on the dynamics of ALS for modeling its prognosis, we are able to provide an overview of the evolution scenarios of the study populations, while at the same time maintaining a detail on the characteristics of each trajectory, also referring to the features of the patients that followed each path.

From a methodological point of view, the analysis we performed integrates statistical inference in the Process Discovery algorithms, allowing a better characterization of the patients’ trajectories and a statistical significance based on standard inferential tests for the observed differences.

A major benefit of the proposed PM-based approach is the graphical representation, that allows to establish a common ground to communicate with experts from different disciplines, and that has been leveraged to analyze the results of this study. As recently stated by Munoz-Gama et al. in a sort of *Process Mining for Healthcare Manifesto*, two of the pillars of analytics in healthcare actually consist in the understandability of what is being done, and the involvement of a heterogeneous team of experts to gather and exploit as much knowledge as possible [52]. Related to this, the attempt to integrate the inferential analysis in our study helps to bridge the gap between what is needed and what is offered.

The use we did of data coming from two distinct sources deserves some considerations: on the one hand, we used PRO-ACT that consists of 23 aggregated clinical trial datasets, on the other we employed for a comparison of the trajectories the ALS–BS dataset that comprehends data of patients followed up in two tertiary clinical centers. As previously reported in literature [46], patients enrolled in clinical trials may differ from epidemiologic cohorts, with the former being mainly characterized by a younger age, a larger diagnostic delay, a longer tracheostomy-free survival, and being more likely men and with a spinal onset. For this reason, differences between the two included cohorts were expected and their possible impact in term of bias on the performed analyses merits an appropriate examination.

A first consideration concerns the difference between the cardinalities of the two datasets. Being ALS a rare disease, with an incidence in Europe estimated around 2.2 per 100,000 person-years [53], the reduced number of subjects included in the ALS–BS dataset is justified by the limited recruiting period of the cohort included in this study. This may impact in terms of generalizability on the considerations made on this dataset both in terms of patients’ characterization at baseline and comparison of the trajectories with PRO-ACT. Nevertheless, we believe that an - although limited - comparison of the process models obtained on PRO-ACT with the behaviours observed in a real-world cohort constitutes a first step in the direction of validating the results, as well as a proof that such analysis can allow inspecting the differences that can emerge between cohorts with different origins.

Moreover, by analyzing the baseline characterization of the PRO-ACT and ALS–BS subjects reported in Table 1 we can observe that some differences actually emerge between the two cohorts, especially in terms of recruitment criteria and observational scheduling, as already briefly mentioned. Specifically, we can observe a significant difference in the baseline FVC value and ALSFRS total score variables, which are both higher in the ALS–BS patients. This is possibly due to the fact that in the tertiary centers the patients are more likely to be taken in charge from the time of diagnosis directly, when they might present a weaker symptomatology.

We can also detect that a possible different codification of the onset site variable has been used in the two datasets, with a further level (“Other”) being used in PRO-ACT. This could impact in the performed stratification study, with two slightly differently defined populations being considered when subsetting the population in bulbar versus spinal subjects.

A discrepancy for what concerns the follow-up duration and number of visits also emerges, in line with the expectations. In fact, clinical trials patients are generally observed over a shorter time window, but more frequently. This might also be reflected in the higher percentage of censored subjects in PRO-ACT. With respect to the disease progression trajectories derived in our study, this may have a dual effect: first, the paths of the PRO-ACT subjects might be shorter in terms of length of the traces (or, in other words, in the number of observed events per patient). This fact finds correspondence in the data, with the PRO-ACT traces reporting a mean number of recorded MiToS impairments per patient equal to 0.91 (median 1, IQR 0–1) versus the 1.48 (median 1, IQR 1–2) of the ALS–BS. This can impact the analysis by limiting the number and the length of common trajectories between the two datasets. In terms of DFG, this can be one of the reasons (together with the limited cardinality of the ALS–BS dataset) why not all the transitions might be observed in both the datasets; in terms of CFM, we would observe a different length of the most common paths. Second, the time of each event could be expected to be more precise in the PRO-ACT cohort due to the higher resolution of the visits. It is interesting observing how the analysis of the kernel density estimations of the (both direct and indirect) transitions examined in Fig. 5 actually reports median times that are longer for the ALS–BS subjects. This might be due to a small delay in the recording of the change of status for the ALS–BS patients indeed, as well as to a slightly increased quality of survival for the real-world subjects due to an improvement in the treatments in the last years or in case some of the trials included in PRO-ACT had required the recruitment of more aggressive ALS forms.

It should be emphasized how a process-oriented analysis (here focused on the impairment trajectories), coupled with the comparison of more traditional descriptive statistics (see Table 1), allows for a more extensive comparison of the cohorts. Indeed, this matched approach allowed us not only to investigate the static characteristics (e.g., at the time of enrollment) of the subjects, but also to assess similarities and differences related to the progression of their clinical condition. By selecting as an analytic technique the DFG, moreover, where the comparison of cohorts is supported by the generation of communicative graphical outputs, we experienced a facilitated discussion of the obtained results with a heterogeneous, multidisciplinary team. In general, this ease of communication can lead to faster identification of differences and more rapid formulation of hypotheses about their origins, possibly enabling the implementation of a proactive approach to ensure a better quality of care.

Besides the considerations on the data characteristics, another possible limitation of our approach might consist in the focus of the analyses limited to one main aspect of ALS evolution only, that is, the progressive impairment of the patients’ functional abilities, and the use of just a couple of baseline patient characteristics.

As potential developments of our study, we envisage two main directions: the former pursues the intention of enhancing the clinical knowledge on ALS and targets the creation of a multicentric study involving a higher number of ALS-specialized centers. This would allow the inclusion of more real-world patients and of additional variables, such as vital signs or lab tests, and new events, such as the administration of nutritional or respiratory support, to define new or enriched patient trajectories and to gain a better understanding of the disease progression mechanisms in real-world cohorts. In the practice, this implies reaching a consensus about the data ontology, the pathways we want to focus on and, last but not least, to develop an infrastructure to support a Rapid Learning paradigm to face the challenges of the *data ownership* and the *patient’s privacy* in PM [54]. The second direction concerns a further improvement of the Process Discovery tools, for example by exploring the predictive potential of other clinical variables investigating how they contribute to the evolution of the clinical pathways. Such application through PM is currently mainly unexplored.

We believe this work can provide an original perspective for analyzing how ALS evolves. The mined processes can be exploited as DDSs, indicating the probability of a patient to follow a given path based on his/her characteristics; further, they can allow to simulate the likely evolution of the disease and, in the future, to assess the impact of treatments.

## Data Availability

The Pooled Resource Open-Access ALS Clinical Trials Database (PRO-ACT) dataset is available at the following link: https://ncri1.partners.org/ProACT/Data/Index/1 (registration required). Restrictions apply to the availability of the ALS–BS dataset in order to ensure the patients’ rights to privacy and anonymity and to prevent inappropriate secondary analyses. Real data or subject identity cannot be inferred in any way from the analyses. The data used for this study are available upon reasonable request to the different centers involved in the study.

## Abbreviations

ALS: Amyotrophic lateral sclerosis
ALSFRS: ALS functional rating scale
ALSFRS-R: Revised ALS functional rating scale
CFM: CareFlow Miner
CSV: Comma-separated values
DDS: Decision support system
Deep-SHAP: Deep Shapley additive explanations
DFG: Directly-Follows Graph
DREAM: Dialogue for Reverse Engineering Assessments and Methods
EL: Event log
FVC: Forced vital capacity
KM: Kaplan–Meier
k-NN: k-nearest neighbours
MiToS: Milano–Torino staging
NeMO: NEuroMuscolar Omnicenter
NIV: Non-invasive ventilation
PM: Process Mining
PRO-ACT: Pooled Resource Open-Access ALS Clinical Trials
RF: Random forests
SPM: Sequential pattern mining

## Footnotes

1 https://nctu.partners.org/proact (download time: March, 2021)

## References

[1] Beghi E, Chiò A, Couratier P, Esteban J, Hardiman O, Logroscino G, Millul A, Mitchell D, Preux P-M, Pupillo E (2011). The epidemiology and treatment of ALS: focus on the heterogeneity of the disease and critical appraisal of therapeutic trials. Amyotroph Lateral Scler.

[2] Goyal NA, Berry JD, Windebank A, Staff NP, Maragakis NJ, van den Berg LH, Genge A, Miller R, Baloh RH, Kern R, Gothelf Y, Lebovits C, Cudkowicz M (2020). Addressing heterogeneity in amyotrophic lateral sclerosis CLINICAL TRIALS. Muscle Nerve.

[3] Küffner R, Zach N, Norel R, Hawe J, Schoenfeld D, Wang L, Li G, Fang L, Mackey L, Hardiman O (2015). Crowdsourced analysis of clinical trial data to predict amyotrophic lateral sclerosis progression. Nat Biotechnol.

[4] Kueffner R, Zach N, Bronfeld M, Norel R, Atassi N, Balagurusamy V, Di Camillo B, Chio A, Cudkowicz M, Dillenberger D (2019). Stratification of amyotrophic lateral sclerosis patients: a crowdsourcing approach. Sci Rep.

[5] Grollemund V, Le Chat G, Secchi-Buhour M-S, Delbot F, Pradat-Peyre J-F, Bede P, Pradat P-F (2021). Manifold learning for amyotrophic lateral sclerosis functional loss assessment. J Neurol.

[6] Westeneng H-J, Debray TP, Visser AE, van Eijk RP, Rooney JP, Calvo A, Martin S, McDermott CJ, Thompson AG, Pinto S (2018). Prognosis for patients with amyotrophic lateral sclerosis: development and validation of a personalised prediction model. Lancet Neurol.

[7] Marin B, Couratier P, Arcuti S, Copetti M, Fontana A, Nicol M, Raymondeau M, Logroscino G, Preux PM (2016). Stratification of ALS patients’ survival: a population-based study. J Neurol.

[8] Taylor AA, Fournier C, Polak M, Wang L, Zach N, Keymer M, Glass JD, Ennist DL (2016). Consortium PRO-A.A.C.T.: predicting disease progression in amyotrophic lateral sclerosis. Ann Clin Transl Neurol.

[9] Carreiro AV, Amaral PM, Pinto S, Tomás P, de Carvalho M, Madeira SC (2015). Prognostic models based on patient snapshots and time windows: predicting disease progression to assisted ventilation in amyotrophic lateral sclerosis. J Biomed Inform.

[10] Müller M, Gromicho M, de Carvalho M, Madeira SC (2021). Explainable models of disease progression in ALS: Learning from longitudinal clinical data with recurrent neural networks and deep model explanation. Comput Methods Programs Biomed Update.

[11] Lundberg SM, Lee SI. A unified approach to interpreting model predictions. Adv Neural Inf Process Syst. 2017;30

[12] Tavazzi E, Daberdaku S, Zandonà A, Vasta R, Nefussy B, Lunetta C, Mora G, Mandrioli J, Grisan E, Tarlarini C et al. Predicting functional impairment trajectories in amyotrophic lateral sclerosis: a probabilistic, multifactorial model of disease progression. J Neurol. 2022;1–21 10.1007/s00415-022-11022-0 PMC9217910 35266043

[13] Carreiro AV, Pinto S, de Carvalho M, Madeira SC, Antunes C. Classification of clinical data using sequential patterns: a case study in amyotrophic lateral sclerosis. In: 2nd workshop on data mining in healthcare and medicine, at SIAM International Conf on Data Mining; 2013.

[14] Zaki MJ, Meira W, Meira W (2014). Data mining and analysis: fundamental concepts and algorithms.

[15] Martins AS, Gromicho M, Pinto S, de Carvalho M, Madeira SC. Learning prognostic models using diseaseProgression patterns: predicting the need forNon-invasive ventilation in amyotrophic lateralSclerosis. IEEE/ACM Trans Comput Biol Bioinf. 2021;19(5):2572–2583. 10.1109/TCBB.2021.3078362 33961562

[16] Gomeni R, Fava M (2014). Pooled resource open-access ALS clinical trials consortium: amyotrophic lateral sclerosis disease progression model. Amyotroph Lateral Scler Frontotemporal Degener.

[17] Cedarbaum JM, Stambler N, Malta E, Fuller C, Hilt D, Thurmond B, Nakanishi A, Group BAS complete listing of the BDNF Study Group, A. The ALSFRS-R: a revised ALS functional rating scale that incorporates assessments of respiratory function. J Neurol Sci. 1999;169(1-2):13–21 10.1016/s0022-510x(99)00210-5 10540002

[18] Ackrivo J, Hansen-Flaschen J, Wileyto EP, Schwab RJ, Elman L, Kawut SM. Development of a prognostic model of respiratory insufficiency or death in amyotrophic lateral sclerosis. Eur Respir J. 2019;53(4). 10.1183/13993003.02237-2018 PMC6684229 30728207

[19] Thakore NJ, Lapin BR, Kinzy TG, Pioro EP (2018). Deconstructing progression of amyotrophic lateral sclerosis in stages: a Markov modeling approach. Amyotroph Lateral Scler Frontotemporal Degener.

[20] Tavazzi E, Daberdaku S, Vasta R, Calvo A, Chiò A, Di Camillo B (2020). Exploiting mutual information for the imputation of static and dynamic mixed-type clinical data with an adaptive k-nearest neighbours approach. BMC Med Inform Decis Mak.

[21] van der Aalst W, Adriansyah A. Process mining manifesto. In: International conference on business process management, Springer; 2011. pp. 169–194.

[22] van der Aalst W, Weijters T, Maruster L (2004). Workflow mining: discovering process models from event logs. IEEE Trans Knowl Data Eng.

[23] van der Aalst W, Adriansyah A, van Dongen B (2012). Replaying history on process models for conformance checking and performance analysis. Wiley Interdiscip Rev Data Min Knowl Discov.

[24] van der Aalst W. Process mining: discovery, conformance and enhancement of business processes. 2011;136. 10.1007/978-3-642-19345-3.

[25] Lenz R, Reichert M (2007). IT support for healthcare processes-premises, challenges, perspectives. Data Knowl Eng.

[26] Rojas E, Munoz-Gama J, Sepúlveda M, Capurro D (2016). Process mining in healthcare: a literature review. J Biomed Inform.

[27] De Roock E, Martin N. Process mining in healthcare–an updated perspective on the state of the art. J Biomed Inform. 2022;103995 10.1016/j.jbi.2022.103995 35077900

[28] Kusuma G, Hall M, Johnson O (2018). Process mining in cardiology: a literature review. Int. J. Biosci. Biochem. Bioinform..

[29] Balakhontceva MA, Funkner AA, Semakova AA, Metsker OG, Zvartau NE, Yakovlev AN, Lutsenko AE, Kovalchuk SV (2018). Holistic modeling of chronic diseases for recommendation elaboration and decision making. Procedia Comput Sci.

[30] Kurniati AP, Johnson O, Hogg D, Hall G. Process mining in oncology: a literature review. In: 2016 6th international conference on information communication and management (ICICM), IEEE; 2016. pp. 291–297.

[31] Dagliati A, Tibollo V, Cogni G, Chiovato L, Bellazzi R, Sacchi L (2018). Careflow mining techniques to explore type 2 diabetes evolution. J Diabetes Sci Technol.

[32] Tavazzi E, Gerard CL, Michielin O, Wicky A, Gatta R, Cuendet MA. A process mining approach to statistical analysis: application to a real-world advanced melanoma dataset. In: International conference on process mining, Springer; 2020. pp. 291–304.

[33] Williams R, Rojas E, Peek N, Johnson OA (2018). Process mining in primary care: a literature review. Stud Health Technol Inform.

[34] Litchfield I, Hoye C, Shukla D, Backman R, Turner A, Lee M, Weber P (2018). Can process mining automatically describe care pathways of patients with long-term conditions in UK primary care? a study protocol. BMJ Open.

[35] Martinez-Millana A, Lizondo A, Gatta R (2019). Process mining dashboard in operating rooms: analysis of staff expectations with analytic hierarchy process. Int J Environ Res Public Health.

[36] Mertens S, Gailly F, Van Sassenbroeck D, Poels G. Integrated declarative process and decision discovery of the emergency care process. Inf Syst Front. 1–23 (2020)

[37] Yang W-S, Hwang S-Y (2006). A process-mining framework for the detection of healthcare fraud and abuse. Expert Syst Appl.

[38] Huang H, Jin T, Wang J. Extracting clinical-event-packages from billing data for clinical pathway mining. In: International conference on smart health, Springer; 2016. pp. 19–31.

[39] Gerhardt R, Valiati JF, dos Santos JVC (2018). An investigation to identify factors that lead to delay in healthcare reimbursement process: a Brazilian case. Big Data Res.

[40] Weijters A, van der Aalst WM, De Medeiros AA. Process mining with the heuristics miner-algorithm. Technische Universiteit Eindhoven, Tech. Rep. WP; 2006. 166, pp. 1–34.

[41] Leemans SJ, Fahland D, van der Aalst WM. Discovering block-structured process models from event logs-a constructive approach. In: International conference on applications and theory of petri nets and concurrency, Springer, 2013. pp. 311–329.

[42] Chiò A, Hammond ER, Mora G, Bonito V, Filippini G (2015). Development and evaluation of a clinical staging system for amyotrophic lateral sclerosis. J Neurol Neurosurg Psychiatry.

[43] Günther CW, van der Aalst WM. Fuzzy mining–adaptive process simplification based on multi-perspective metrics. In: International conference on business process management, Springer; 2007. pp. 328–343.

[44] Gatta R, Vallati M, Lenkowicz J, Rojas E, Damiani A, Sacchi L, De Bari B, Dagliati A, Fernandez-Llatas C, Montesi M, Marchetti A, Castellano M, Valentini V. Generating and comparing knowledge graphs of medical processes using pminer. 2017. 10.1145/3148011.3154464.

[45] Atassi N, Berry J, Shui A, Zach N, Sherman A, Sinani E, Walker J, Katsovskiy I, Schoenfeld D, Cudkowicz M (2014). The PRO-ACT database design, initial analyses, and predictive features. Neurology.

[46] Chiò A, Canosa A, Gallo S, Cammarosano S, Moglia C, Fuda G, Calvo A, Gabriele M (2011). ALS clinical trials: Do enrolled patients accurately represent the ALS population?. Neurology.

[47] ALS CNTF treatment study (ACTS) phase I-II study group: the amyotrophic lateral sclerosis functional rating scale: assessment of activities of daily living in patients with amyotrophic lateral sclerosis. Arch Neurol. 1996; 53(2): 141–147. 10.1001/archneur.1996.00550020045014 8639063

[48] Voustianiouk A, Seidel G, Panchal J, Sivak M, Czaplinski A, Yen A, Appel SH, Lange DJ (2008). ALSFRS and appel ALS scores: discordance with disease progression. Muscle Nerve Off J Am Assoc Electrodiagn Med.

[49] Wicks P, Massagli M, Wolf C, Heywood J (2009). Measuring function in advanced ALS: validation of ALSFRS-EX extension items. Eur J Neurol.

[50] Carvalho MD, Swash M (2009). Awaji diagnostic algorithm increases sensitivity of EL escorial criteria for ALS diagnosis. Amyotroph Lateral Scler.

[51] Chio, A., Logroscino, G., Hardiman, O., Swingler, R., Mitchell, D., Beghi, E., Traynor, B.G., Consortium, E., (2009). Prognostic factors in ALS: a critical review. Amyotroph Lateral Scler.

[52] Munoz-Gama J, Martin N, Fernandez-Llatas C, Johnson OA, Sepúlveda M, Helm E, Galvez-Yanjari V, Rojas E, Martinez-Millana A, Aloini D et al. Process mining for healthcare: characteristics and challenges. J Biomed Inform. 2022;103994. 10.1016/j.jbi.2022.103994 35104641

[53] Logroscino G, Piccininni M (2019). Amyotrophic lateral sclerosis descriptive epidemiology: the origin of geographic difference. Neuroepidemiology.

[54] Gatta R, Vallati M, Lenkowicz J, Masciocchi C, Cellini F, Boldrini L, Fernandez Llatas C, Valentini V, Damiani A. On the feasibility of distributed process mining in healthcare. In: International conference on computational science, Springer; 2019. pp. 445–452.

